# Head-to-Head Comparison of Etest, MICRONAUT-AM EUCAST and Reference Broth Microdilution-Based CLSI Results for *Candida kefyr* Antifungal Susceptibility Testing: Implications for Detection of Reduced Susceptibility to Amphotericin B

**DOI:** 10.3390/jof11080570

**Published:** 2025-07-30

**Authors:** Mohammad Asadzadeh, Suhail Ahmad, Jacques F. Meis, Josie E. Parker, Wadha Alfouzan

**Affiliations:** 1 Department of Microbiology, Faculty of Medicine, Kuwait University, Jabriya 46300, Kuwait; mohammad.assadzadeh@ku.edu.kw (M.A.);; 2 Department of Medical Microbiology, Radboud University Medical Center, 6525 Nijmegen, The Netherlands; jacques.meis@gmail.com; 3 Center of Expertise in Mycology Radboudumc/Canisius Wilhelmina Hospital, 6532 Nijmegen, The Netherlands; 4 Department I of Internal Medicine and Excellence Center for Medical Mycology, Faculty of Medicine, University Hospital Cologne, 50937 Cologne, Germany; 5 Molecular Biosciences Division, School of Biosciences, Cardiff University, Cardiff CF10 3AT, UK; parkerj21@cardiff.ac.uk

**Keywords:** *Candida kefyr*, antifungal susceptibility testing, Etest, MICRONAUT-AM assay, reference CLSI method, comparative performance

## Abstract

Invasive infections with rare yeasts are increasing worldwide and are associated with higher mortality rates due to their resistance to antifungal drugs. Accurate antifungal susceptibility testing (AFST) is crucial for proper management of rare yeast infections. We performed AFST of 74 *Candida kefyr* isolates by Etest, EUCAST-based MICRONAUT-AM assay (MCN-AM) and reference Clinical and Laboratory Standards Institute broth microdilution method (CLSI). Essential agreement (EA, ±1 two-fold dilution), categorical agreement (CA), major errors (MEs) and very-major errors (VmEs) were determined using epidemiological cut-off values of ≤1.0 µg/mL, ≤0.03 µg/mL, ≤0.5 µg/mL and ≤1 µg/mL, defining wild-type isolates for fluconazole, voriconazole, micafungin and amphotericin B (AMB), respectively. Results for AMB susceptibility were correlated with *ERG2/ERG3* mutations and total-cell sterols. CA of ≥97% was recorded between any two methods while EA varied between 72 and 82%, 87 and 92%, and 49 and 76% for fluconazole, voriconazole and micafungin, respectively. For AMB, CAs between CLSI and Etest; CLSI and MCN-AM; MCN-AM and Etest were 95% (4 ME, 0 VmE), 96% (3 ME, 0 VmE) and 99%, respectively, while EA varied from 32% to 69%. Non-synonymous *ERG2/ERG3* mutations and no ergosterol were found in seven of eight isolates of non-wild types for AMB by Etest. Our data show that Etest, CLSI and MCN-AM methods are suitable for AFST of *C. kefyr* for fluconazole, voriconazole and micafungin. Excellent CAs for AMB between Etest and MCN-AM with concordant sterol profiles but not with CLSI suggest that Etest is also an excellent alternative for the detection of *C. kefyr* isolates with reduced susceptibility to AMB.

## 1. Introduction

The incidence of invasive fungal infections (IFIs) is gradually increasing worldwide due to the increasing population of at-risk patients [1,2]. *Candida*/yeast species are an important cause of IFIs [3]. Colonization of skin and mucosal surfaces of respiratory/gastrointestinal/urogenital tracts predispose hospitalized patients to invasive infections by *Candida*/yeast species [4,5,6]. The epidemiology of *Candida*/yeast infections has also changed during the last few decades. The majority (>50%) of invasive yeast infections are now caused by non-*albicans* species of *Candida* or other yeast species [7,8]. The increasing use of antifungal prophylaxis has also resulted in breakthrough invasive infections in susceptible patients by drug-resistant/multidrug-resistant *Candida*/yeast species [9,10,11,12]. Rapid diagnosis and accurate antifungal susceptibility testing (AFST) of infrequently encountered yeast species is critical for proper management of patients with invasive infections [13,14,15,16].

*Candida kefyr* (now known as *Kluyveromyces marxianus*) is an uncommon yeast from clinical specimens including blood and an emerging pathogen infecting immunocompromised patients, particularly those with hematological malignancies pre-exposed to antifungal drugs [12,17,18,19,20]. We recently performed AFST of a large number of clinical *C. kefyr* isolates by Etest and identified several isolates exhibiting reduced susceptibility to amphotericin B (AMB) [21]. However, when 12 selected isolates were also tested by the colorimetric SensiTitre Yeast One (SO), only 10 isolates yielded concordant results (categorical agreement, CA of 83.3%) while 2 wild-type (WT) isolates by Etest for AMB were scored as non-WT by SO. The essential agreement (EA, ±2 two-fold dilutions) between Etest and SO was also poor (7 of 12 or 58.3%). Previous studies have also shown poor concordance between different AFST methods of AMB for some yeast species, particularly when AFST is performed by the rapid commercial systems [22,23,24,25,26,27,28,29]. Since accurate AFST of rare yeast isolates is essential for proper management of invasive infections [12,14,16], this study performed head-to-head comparison of AFST results obtained by Etest, microdilution-based colorimetric MICRONAUT-AM EUCAST assay and reference broth microdilution-based Clinical and Laboratory Standard Institute (CLSI) method for clinical *C. kefyr* isolates and corelated AMB AFST data with alterations in *ERG2/ERG3* genes and total cell ergosterol levels.

## 2. Materials and Methods

### 2.1. Reference Strains and Clinical C. kefyr Isolates

*Candida kefyr* ATCC28838, *C. albicans* ATCC90028 and *Candida parapsilosis* ATCC22019 were used as references. A total of 74 clinical *C. kefyr* isolates cultured from 74 patients were used. These isolates originated from urine (*n* = 36), sputum (*n* = 15), blood (*n* = 4), abdominal/peritoneal fluid (*n* = 4), bronchoalveolar lavage (*n* = 3), endotracheal secretion (*n* = 3), tracheal aspirate (*n* = 2), vaginal swab (*n* = 2) and one isolate each from ear swab, gastric aspirate, throat swab, tissue biopsy and an unknown source. The clinical specimens were collected from patients hospitalized in various government hospitals across Kuwait after obtaining verbal consent only as part of routine patient care and diagnostics for detection and AFST of fungal pathogens. The clinical details, treatment given and outcome were not available for the patients from whom *C. kefyr* isolates were obtained. The specimens were processed in BACTEC Plus blood culture bottles (Beckton Dickinson, Sparks, MD, USA) and/or on Sabouraud dextrose agar (SDA) (Difco) supplemented with chloramphenicol (50 µg/mL) plates as described previously [30]. All isolates were identified to the species level by the Vitek 2 yeast identification system (bioMérieux, Marcy-l’Etoile, France) as well as by PCR amplification and PCR sequencing of the internal transcribed spacer (ITS) region of rDNA as described previously [20].

### 2.2. Antifungal Susceptibility Testing

The AFST of clinical *C. kefyr* isolates against fluconazole, voriconazole, micafungin and amphotericin B was performed by three different methods: Etest, microdilution-based colorimetric MICRONAUT-AM EUCAST assay (MCN-AM) and reference broth microdilution-based Clinical and Laboratory Standard Institute method (CLSI). *Candida kefyr* ATCC28838, *C. parapsilosis* ATCC22019 and *C. albicans* ATCC90028 were used for quality control. The Etest strips contain an impregnated concentration gradient of specific antifungal drugs (bioMérieux SA, Marcy-l’-Etoile, France) and the testing was performed according to the manufacturer’s instructions and as described previously [20]. The MCN-AM (Merlin Diagnostica GmbH, Bornheim, Germany) microtiter plate wells supplied by the company contained a lyophilized concentration gradient of various antifungals, which were reconstituted according to the instructions supplied by the manufacturer. The test was performed and the results were interpreted by following the manufacturer’s instructions and according to the European Committee on Antimicrobial Susceptibility Testing (EUCAST) method. The plates were read spectrophotometrically after 24 h of incubation at 35 °C and the minimum inhibitory concentration (MIC) values were determined as the drug concentration that inhibited 50% of the growth for all drugs except amphotericin B for which >90% growth inhibition was used [31,32]. The reference broth microdilution-based CLSI method was performed according to the M27A protocol guidelines by using pure, laboratory-grade antifungal drugs (Sigma-Aldrich, Athens, Greece). The microtiter plates were incubated at 35 °C, the wells were observed visually after 24 h and the MICs were recorded as the lowest drug concentrations yielding total inhibition of visual growth compared to growth control well, as described previously [33]. Since susceptibility breakpoints are not available for *C. kefyr*, epidemiological cut-off values were used for interpreting the MIC values for fluconazole, voriconazole, micafungin and AMB. Isolates with an MIC of ≤1 µg/mL, ≤0.03 µg/mL, ≤0.5 µg/mL and ≤1 µg/mL were considered as wild-type (WT) isolates, while isolates with MICs of >1 µg/mL, >0.03 µg/mL, >0.5 µg/mL and >1 µg/mL were considered as non-wild-type (non-WT) isolates for fluconazole, voriconazole, micafungin and AMB, respectively [34,35]. Repeat AFST was performed on the isolates yielding discrepant susceptibility results by any of the three methods.

### 2.3. Sequencing of ERG Genes and Total Cell Ergosterol Analyses

The *ERG2*, *ERG3* and *ERG6* genes involved in ergosterol biosynthesis are mutated in some yeasts, exhibiting reduced susceptibility to AMB [24,36,37,38,39,40,41]. The nucleotide and amino acid coding sequences of *ERG2*, *ERG3* and *ERG6* genes were determined by PCR-sequencing for all isolates non-WT for AMB by any of the three AST methods and a few WT isolates. Each *ERG* gene was amplified by using gene-specific primers and the amplicons were bidirectionally sequenced by using internal primers, as described previously [21]. The DNA sequence data for the ITS region of rDNA, *ERG2*, *ERG3* and *ERG6* genes for selected *C. kefyr* isolates were submitted to GenBank under accession no. OQ520304 to OQ520311 and OQ542694 to OQ542744. The ergosterol and other sterol contents in yeast cells of selected *C. kefyr* isolates were also determined by gas chromatography–mass spectrometry (GC-MS) as described previously [21,24]. Briefly, *C. kefyr* isolates were grown in Roswell Park Memorial Institute (RPMI) medium (pH 7.0) (Sigma-Aldrich Inc., St. Louis, MO, USA) for 24 h at 37°, cells were harvested and washed three times with sterile water and the non-saponifiable lipids were extracted by using alcoholic KOH. Samples, dried in a vacuum centrifuge, were derivatized with trimethylsilane (TMS), the TMS-derivatized sterols were analyzed by using gas chromatography–mass spectrometry (GC-MS) (Thermo 1300 GC coupled to a Thermo ISQ mass spectrometer, Thermo Scientific, Loughborough, UK) and identified with reference to retention times and fragmentation spectra for known standards. The data were analyzed by using Xcalibur software (version 3.1, Thermo Scientific) to determine sterol profiles and the results of three replicates from each sample were used to calculate the mean percentage ± standard deviation for each sterol, as described previously [21,24].

### 2.4. Sequencing of Hotspot-1 and Hotspot-2 Regions of FKS1 Gene

The hotspot-1 and hotspot-2 regions of *FKS1* gene, often mutated in echinocandin resistant yeast species, were amplified by using *C. kefyr*-specific CkefFKS1F1 + CkefFKS1R1 and CkefFKS1F2 + CkefFKS1R2 primers, respectively, as described previously [20]. The amplicons were purified and were sequenced bidirectionally by using internal sequencing primer CkefFKS1F1S or CkefFKS1R1S for hotspot-1 and CkefFKS1F2S or CkefFKS1R2S for hotspot-2, as described previously [20].

### 2.5. Fingerprinting of C. kefyr Isolates

The phylogenetic relationship among *C. kefyr* isolates non-WT for AMB and three randomly selected WT isolates was determined by comparing concatenated sequence data of *ERG2*, *ERG*3 and *ERG6* genes together with the ITS region of rDNA. The DNA sequence data from *C. kefyr* ATCC28838 were used as a reference. Multiple sequence alignments were performed with Clustalw muscle (https://www.ebi.ac.uk/Tools/msa/muscle/, accessed on 5 May 2025) and phylogenetic analysis was performed with Molecular Evolutionary Genetic Analysis (MEGA) version 6 software using the maximum likelihood method and bootstrap analysis with 1000 replicates, as described previously [24].

### 2.6. Statistical Analysis

Statistical analysis was performed by using Fisher’s exact test and probability levels < 0.05 by the two-tailed test were considered as significant. Statistical analyses were performed by using WinPepi software ver. 11.65 (PEPI for Windows, Microsoft Inc., Redmond, WA, USA). The strength of agreement between results by different AFST methods was determined using the GraphPad Prism software version 10.5.0.774 (GraphPad, La Jolla, CA, USA) and a Kappa coefficient (κ) value of 0–0.2, 0.21–0.4, 0.4–0.6, 0.61–0.8 and 0.81–1 indicated poor, fair, moderate, substantial and almost perfect agreement, respectively.

## 3. Results

### 3.1. Clinical Isolates and Distribution of MIC Values by CLSI, Etest and MCN-AM Tests

Most of the clinical *C. kefyr* isolates used in this study were cultured from urine (49%) and respiratory tract (32%) specimens from patients hospitalized in various hospitals across Kuwait. The MIC values obtained for the reference *C. albicans*, *C. parapsilosis* and *C. krusei* strains used as quality control were within the recommended range for all four antifungal drugs and by all the three testing methods. The MIC values obtained for the 74 *C. kefyr* isolates for fluconazole, voriconazole, micafungin and AMB by the three testing methods are presented in Table 1. For fluconazole, the largest and smallest variations in MIC values were obtained by the Etest and CLSI method, respectively. All three methods detected two (but not the same) isolates as non-WT for fluconazole. For voriconazole, little variation in MIC values was obtained by all three methods and only one isolate was detected as non-WT by Etest and MCN-AM (Table 1). For micafungin, the MIC values of all isolates were nearly comparable and only one isolate was detected as non-WT by Etest. However, similar to previously reported data [20], no non-synonymous mutation was detected in the hotspot-1 or hotspot-2 region of the *FKS1* gene in the isolate (Kw2153/18) scored as non-WT for micafungin by Etest. For AMB, the largest variation in MIC values was obtained by Etest followed by CLSI, while the majority (42 of 74, 57%) of isolates yielded the epidemiological cutoff MIC value (1 µg/mL) by the MCN-AM. However, eight isolates were scored as non-WT by Etest. Interestingly, seven and four of these isolates were also detected as non-WT by MCN-AM and CLSI methods. Repeat AFST performed on isolates yielding discrepant results by any of the three methods exhibited the same pattern of susceptibility with little or no variation in the MIC values as that obtained during the first tests. 

### 3.2. Performance Comparison of CLSI, Etest and MCN-AM AFST Results

The MIC range, modal values, CA and EA based on ±1 two-fold dilution and ±2 two-fold dilution for the four antifungal drugs for 74 *C. kefyr* isolates by CLSI versus Etest, CLSI versus MCN-AM and Etest versus MCN-AM are presented in Table 2. Although MIC values of fluconazole, voriconazole and micafungin varied considerably by the three methods, the modal MIC values were nearly similar and the CA was also very high (≥97%) with none or only one major error and/or very major error between the reference CLSI and the other two methods (Table 1). The kappa values of 0.486, 0.486 and 1 also indicated moderate agreement, moderate agreement and perfect agreement between CLSI versus Etest, CLSI versus MCN-AM and Etest versus MCN-AM, respectively, for fluconazole. The EA at ±1 two-fold dilution was also high for fluconazole (72% to 82%) and voriconazole (87% to 92%), which increased from 89% to 97% for fluconazole and 99% to 100% for voriconazole at ±2 two-fold dilution. Interestingly, the EA was lowest for CLSI versus Etest for fluconazole at both ±1 two-fold dilution and ±2 two-fold dilution values. On the contrary, the EA for micafungin was the highest for CLSI versus Etest at both ±1 two-fold dilution (87%) and ±2 two-fold dilution (100%) (Table 2). The CA for AMB between CLSI versus Etest was only 95% (4 major errors) and between CLSI versus MCN-AM was 96% (3 major errors) while it was 99% between Etest versus MCN-AM. The kappa values of 0.641, 0.707 and 0.926 also indicated substantial agreement, substantial agreement and almost perfect agreement between CLSI versus Etest, CLSI versus MCN-AM and Etest versus MCN-AM, respectively, for AMB. The EA for AMB was lower for ±1 two-fold dilutions as it varied from 32% to 69% but improved considerably for ±2 two-fold dilution values (73% to 88%) (Table 2). The EA for AMB was the highest for CLSI versus Etest at ±1 two-fold dilution (69%) and for CLSI versus MCN-AM at ±2 two-fold dilution (88%) (Table 2). Collectively, 66 and 4 isolates were detected as WT and non-WT, respectively, by all three methods, while the remaining 4 isolates were detected as non-WT for AMB by one or both AFST methods.

**Figure 1:**
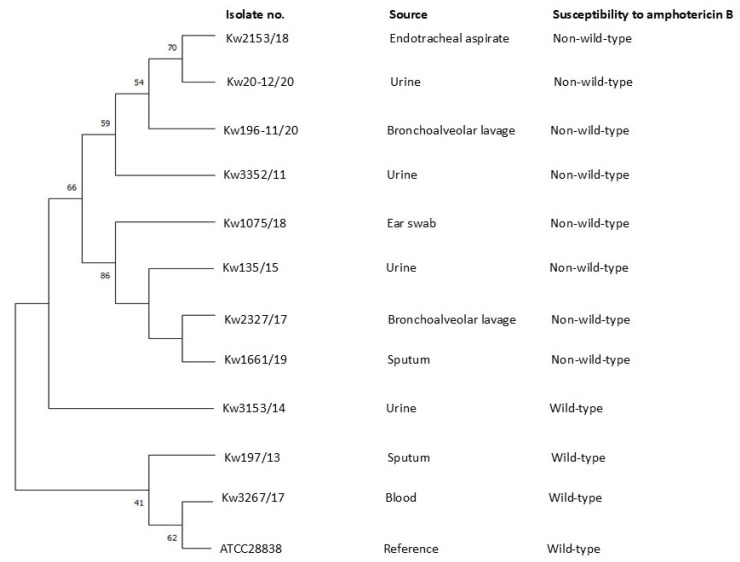
Maximum likelihood phylogenetic tree based on concatenated sequence data for the ITS region of rDNA, *ERG2*, *ERG3* and *ERG6* genes from 11 *C. kefyr* isolates from Kuwait together with reference *C. kefyr* ATCC28838. The numbers on the node branches are bootstrap frequencies from 1000 replicates.

The phylogenetic relationship among 11 selected *C. kefyr* isolates was also determined by comparing concatenated sequence data of ITS region of rDNA, *ERG2*, *ERG*3 and *ERG6* genes. The phylogenetic tree showed that all eight isolates detected as non-WT for AMB by one or more methods were genotypically distinct strains (Figure 1).

### 3.3. Discordant AMB AFST Results, ERG Gene Sequences and Ergosterol/Fecosterol Levels

The discordant MIC results by the three AFST methods for the eight non-WT isolates for AMB by Etest and three randomly selected WT isolates for AMB by all three methods together with *ERG2/ERG3* gene sequence data and total cell ergosterol/fecosterol levels are presented in Table 3. All three (Kw197/13, Kw3153/14 and Kw3267/17) WT isolates for AMB by all three methods contained wild-type *ERG3* sequences and either wild-type sequence or a non-synonymous mutation (A113S) in *ERG2* that did not abrogate its function and ergosterol as the major (>60%) and fecosterol as minor (<5%) cell sterol (Table 3). All four (Kw135/15, Kw2327/17, Kw1075/18 and Kw20-12/20) non-WT isolates for AMB by all three methods contained wild-type *ERG3* sequences and a non-synonymous mutation in *ERG2,* which abrogated its function as ergosterol was completely absent, while fecosterol and other related sterols (such as Ergosta-8-enol, Ergosta-8,22-dienol and Ergosta-5,8,22-trienol) accumulated (Table 3). Two other (Kw3352/11 and Kw196-11/20) non-WT isolates for AMB by Etest and MCN-AM only also contained a non-synonymous mutation in *ERG2,* which abrogated its function as ergosterol was completely absent, while fecosterol and other related sterols (such as Ergosta-8-enol, Ergosta-8,22-dienol and Ergosta-5,8,22-trienol) accumulated (Table 3). Another (Kw1661/19) non-WT isolate for AMB by Etest and MCN-AM only contained a wild-type *ERG3* sequence and a deletion of one nucleotide (Δ617t) causing frame-shift mutation in *ERG2* near the C-terminal end, which apparently did not affect its function as ergosterol was present, while fecosterol and other related sterols (such as Ergosta-8-enol, Ergosta-8,22-dienol and Ergosta-5,8,22-trienol) were absent or were minor cell sterols (Table 3). The remaining (Kw2153/18) non-WT isolate for AMB by Etest only contained a wild-type *ERG2* sequence and a non-synonymous (S218P) mutation in *ERG*3, which abrogated its function as ergosterol was completely absent, while Ergosta-7,22-dienol accumulated as the major cell sterol (Table 3). The *ERG6* gene sequences were unremarkable as they were either the same as the reference strain or contained polymorphisms not connected with alterations in sterol levels [21].

## 4. Discussion

Rapid diagnosis and accurate antifungal susceptibility testing (AFST) of rare yeast species is critical for proper management of patients with invasive infections [13,14,15,16]. Accurate in vitro AFST results for rare yeasts are usually obtained by using reference CLSI or EUCAST methodologies [42,43,44,45]. However, these procedures are usually not preferred by routine clinical mycology laboratories as they involve labor-intensive and time-consuming protocols compared to simpler and easy-to-use commercial tests such as Vitek 2, SensiTitre Yeast One and Etest [22,28,34,46,47]. Previous studies have shown poor concordance between different AFST methods for some triazoles and AMB but not for echinocandins (anidulafungin and micafungin) for some yeast species, particularly when AFST is performed by the commercial systems [22,23,24,25,26,27,28,29,32,34,35,45,46,47]. The discrepant results have been more striking for the recently emerged fungal pathogen *Candida auris* (now known as *Candidozyma auris*), as nearly 50% of clinical isolates were erroneously classified as AMB-resistant by some AFST methods [26,27,29,48]. Since clinical breakpoints for AMB are not available for many *Candida*/yeast species, method-dependent epidemiological cutoff values have been proposed to overcome these limitations [26,27,28,29,35,45,46].

In this study, we have performed AFST using the reference CLSI and two commercial (Etest and MCN-AM) methods on a large collection of *C. kefyr* isolates (*n* = 74) recovered from a variety of clinical specimens of hospitalized patients in Kuwait. All 74 isolates used in this study were identified as *C. kefyr* by both phenotypic and genotypic methods. The CA (a measure of categorical interpretation of the MIC values as susceptible or susceptible dose-dependent/intermediate or resistant) [49] for micafungin between any two methods was nearly perfect (≥99%), implying that any of the three methods could be used for in vitro AFST for this echinocandin. Furthermore, EA, though only moderate (varying from 49% to 76%) at ±1 two-fold dilutions, was nearly 90% or more at ±2 two-fold dilutions for any two methods. The data are consistent with several previous reports showing good performance of rapid methods for in vitro AFST of yeast species against echinocandins [27,32,34,47,50].

The CA for fluconazole and voriconazole between any two methods was also very high (≥97% for fluconazole) or nearly perfect (≥99% for voriconazole), suggesting that any of the three methods could also be used for in vitro AFST of *C. kefyr* for these two triazole antifungals. Again, the EA for fluconazole at ±1 two-fold dilutions was lower, varying from 72% to 82%, but it was nearly 90% or more at ±2 two-fold dilutions for any two methods, while for voriconazole, it was nearly 90% or more even at ±1 two-fold dilutions. These data also imply that rapid methods such as Etest and MCN-AM assay could be used for accurate in vitro AFST of *C. kefyr* against fluconazole and voriconazole antifungals. Other studies have suggested method-dependent epidemiological cutoff values for detecting resistance to fluconazole and voriconazole by SensiTitre YeastOne and Etest for several *Candida* species [35,46]. On the contrary, other rapid methods such as MCN-AM and Vitek 2 have yielded erroneous AFST results for fluconazole for some yeast species (e.g., *C. auris*) in recent studies [25,27,29].

The lowest CA for any two methods was obtained for AMB. There were four major errors (WT by CLSI but non-WT by Etest) for CLSI and Etest and three major errors (WT by CLSI but non-WT by MCN-AM) for CLSI and MCN-AM assays. The EA was also the lowest for AMB for any two methods at ±1 two-fold dilutions varying from 32% to 69%, which improved to 73% to 88% for ±2 two-fold dilutions. The Etest method yielded the widest ranges while the MCN-AM and CLSI methods yielded lower ranges of MIC values for AMB. Previous studies with some yeast species isolates have also shown that the CLSI method yields a narrow range of AMB MIC values and so is often unable to separate WT isolates from non-WT isolates [23,25,26,27,28,51]. On the other hand, the Etest method has shown better discrimination of WT from non-WT isolates for AMB and in some cases, these data have correlated with outcome of patients treated with AMB [23,25,26]. Of the four (Kw3352/11, Kw2153/18, Kw1661/19 and Kw196-11/20) isolates with discordant results, three (Kw3352/11, Kw1661/19 and Kw196-11/20) non-WT isolates for AMB obtained by Etest were also non-WT for AMB obtained by MCN-AM assay. Previous studies carried out on various *Candida*/yeast species isolates with reduced susceptibility to AMB have shown genetic alterations in *ERG2*, *ERG3* and *ERG6* genes involved in ergosterol biosynthesis with concomitant absence of ergosterol and accumulation of the corresponding ergosterol biosynthesis intermediates in the yeast cell [24,36,37,38,39,40,41]. The AFST data correlated with genetic alterations in *ERG2*, *ERG3* and *ERG6* genes and total cell sterol levels for the three selected *C. kefyr* isolates WT for AMB and all eight non-WT isolates for AMB by one or more methods.

All three (Kw197/13, Kw3153/14 and Kw3267/17) *C. kefyr* isolates determined as WT for AMB by all the three methods contained ergosterol as the main total cell sterol similar to the reference *C. kefyr* isolate (ATCC28838). On the contrary, all four (Kw135/15, Kw2327/17, Kw1075/18 and Kw20-12/20) non-WT isolates for AMB by all three methods lacked ergosterol and accumulated not only fecosterol but also ergosta-8-enol, ergosta-8,22-dienol and ergosta-5,8,22-trienol (cumulatively shown as fecosterol plus in Table 3), indicating loss of ERG2 protein function, a finding similar to that observed with *C. glabrata* isolates exhibiting reduced susceptibility to AMB [24,37]. Two other (Kw3352/11 and Kw196-11/20) non-WT isolates for AMB obtained by Etest and MCN-AM but detected as WT by CLSI also lacked ergosterol and accumulated fecosterol, ergosta-8-enol, ergosta-8,22-dienol and ergosta-5,8,22-trienol (similar to the four isolates mentioned above), indicating loss of ERG2 protein function. These findings suggest that the CLSI erroneously classified these two isolates as WT for AMB while both Etest and MCN-AM correctly classified these isolates as non-WT for AMB. The CLSI method also yielded lower (1 µg/mL) MIC values for many *C. glabrata* isolates that contained *ERG2/ERG6* mutations and lacked ergosterol, while Etest yielded higher (1–4 µg/mL) MIC values supporting lower susceptibility to this antifungal drug [24]. Another non-WT isolate (Kw1661/19) for AMB obtained by Etest and MCN-AM but classified as WT by CLSI was even more interesting. This isolate contained a deletion of one nucleotide (Δ617t) near the C-terminal end of the *ERG2*-encoded protein of 223 amino acids causing a frame shift mutation and creation of a premature termination at codon 208. However, this isolate contained normal ergosterol levels and so the deletion frame shift mutation near the C-terminal end does not appear to have affected the ERG2 protein activity. Thus, detection of ergosterol and other sterol levels in yeast isolates appears to be a more accurate method than genetic screening for the detection of non-WT isolates for AMB [52]. Some recent studies have also reported overestimation of AMB resistance in *C. auris* by rapid commercial Vitek 2 and SensiTitre YeastOne AFST methods [25,26,27].

The remaining (Kw2153/18) isolate, detected as WT for AMB by CLSI and MCN-AM assay but as non-WT by Etest, contained a non-synonymous (S218P) mutation in the *ERG3* gene, lacked ergosterol and accumulated ergosta-7,22-dienol, indicating loss of *ERG3* protein function. Loss of ERG3 protein function has been reported as another mechanism for conferring reduced susceptibility to AMB and impaired virulence in *C. albicans* and some other yeast species [38,40,41,53,54,55]. Molecular fingerprinting studies showed that C. kefyr isolates with reduced susceptibility to AMB were not clonally related.

## 5. Conclusions

The AFST was performed on a large collection (*n* = 74) of *C. kefyr* strains isolated from diverse clinical specimens by the reference CLSI and two (Etest and MCN-AM) rapid, commercial methods. Although the CA of ≥95% was recorded between any two methods for all four drugs, the lowest CAs were obtained for AMB between CLSI and Etest tests; CLSI and MCN-AM tests. Non-synonymous *ERG2/ERG3* mutations and no ergosterol were found in seven of eight isolates non-WT for AMB by Etest. Our data show that Etest, CLSI and MCN-AM methods are suitable for AFST of *C. kefyr* for fluconazole, voriconazole and micafungin. Excellent CAs for AMB between Etest and MCN-AM with concordant sterol profiles but not with CLSI suggest that Etest is also a good alternative for the detection of *C. kefyr* isolates with reduced susceptibility to AMB.

## Figures and Tables

**Table 1:** In vitro antifungal susceptibility testing (AFST) results of *C. kefyr* isolates (n = 74) against fluconazole (FLU), voriconazole (VOR), micafungin (MFG) and amphotericin B (AMB) by reference broth microdilution-based Clinical Laboratory Standard Institute (CLSI), Etest and colorimetric MICRONAUT-AM (MCN-AM) methods.

Antifungal	AST	No. of Isolates with Minimum Inhibitory Concentration (MIC) (µg/mL) of																					
Drug	Method	≤0.01	0.02	0.03	0.05	0.06	0.09	0.13	0.19	0.25	0.38	0.5	0.75	1	2	3	4	8	16	32	64	128	256
FLU	CLSI					4		10		26 *		25		7	**2**								
	Etest			1		1	4	2	17 *	11	10	16	6	4		**1**							**1**
	MCN-AM									62 *		7		3			**1**					**1**	
VOR	CLSI		69 *	5																			
	Etest	43 *	25	5																**1**			
	MCN-AM	72 *		1														**1**					
MFG	CLSI	1	3	31		32 *		5		2													
	Etest	3	21 *	11	15	14	7	2	1					**1**									
	MCN-AM		70 *	3						1													
AMB	CLSI					3		9		51 *		5		2	**4**								
	Etest			3		6	1	2	6	10	19 *	11	5	3			**1**			**7**			
	MCN-AM											25		42 *	**3**		**2**		**2**				

The modal MIC values are shown by an asterisk and MIC values indicative of non-wild-type pattern according to the epidemiological cutoff values are shown in bold face and underlined number.

**Table 2:** Comparison of method-specific minimum inhibitory concentration (MIC) modal values and ranges together with categorical agreement (CA) with major errors (MaEs) and very major errors (vmEs) and essential agreement (EA) for 1 or 2 two-fold dilutions for *C. kefyr* isolates.

Antifungal	AFST Methods	Modal (Range) MIC (µg/mL) Values	Ecoff Value	% CA (MaE, VmE)	% EA	
Drug			(µg/mL)		±1 Two-Fold	±2 Two-Fold
FLU	CLSI vs. Etest	0.25 (0.06–2) vs. 0.19 (0.03–256)	1	97% (1, 1)	72%	89%
	CLSI vs. MCN-AM	0.25 (0.06–2) vs. 0.25 (0.25–128)	1	97% (1, 1)	82%	95%
	Etest vs. MCN-AM	0.25 (0.25–128) vs. 0.19 (0.03–256)	1	100% (N/A)	80%	97%
VOR	CLSI vs. Etest	0.02 (0.02–0.03) vs. 0.01 (0.01–32)	0.03	99% (1, 0)	91%	99%
	CLSI vs. MCN-AM	0.02 (0.02–0.03) vs. 0.01 (0.01–8)	0.03	99% (1, 0)	92%	99%
	Etest vs. MCN-AM	0.01 (0.01–8) vs. 0.01 (0.01–32)	0.03	100% (N/A)	87%	100%
MFG	CLSI vs. Etest	0.06 (0.01–0.25) vs. 0.02 (0.01–1)	0.5	99% (1, 0)	76%	99%
	CLSI vs. MCN-AM	0.06 (0.01–0.25) vs. 0.02 (0.02–0.25)	0.5	100% (0, 0)	49%	89%
	Etest vs. MCN-AM	0.02 (0.02–0.25) vs. 0.02 (0.01–1)	0.5	99% (N/A)	49%	88%
AMB	CLSI vs. Etest	0.25 (0.06–2) vs. 0.38 (0.03–32)	1	95% (4, 0)	69%	85%
	CLSI vs. MCN-AM	0.25 (0.06–2) vs. 1 (0.5–16)	1	96% (3, 0)	32%	88%
	Etest vs. MCN-AM	1 (0.5–16) vs. 0.38 (0.03–32)	1	99% (N/A)	45%	73%

FLU, fluconazole; VOR, voriconazole; MFG, micafungin; AMB, amphotericin B; CLSI, reference broth microdilution-based Clinical Laboratory Standard Institute method, MCN-AM, colorimetric MICRONAUT-AM method; N/A, not applicable.

**Table 3:** Discordant MIC results by the three AFST methods for 11 *C. kefyr* isolates and reference *C. kefyr* ATCC28838 together with *ERG2/ERG3* gene sequence data and total cell ergosterol/fecosterol/other major sterol levels.

Patient	Source ^a^	Isolate	CLSI MIC	Etest MIC	MCN-AM MIC	*ERG2*	*ERG3*	Total Cell Sterol Detected as (%) ^e^			
No.		No.	for AMB ^b^	for AMB ^b^	for AMB ^b^	Sequence ^c^	Sequence ^c^	Ergosterol	Fecosterol	Fecosterol Plus ^f^	Ergosta-7,22-dienol
N. A.	Human	ATCC28838	0.25	0.25	0.5	WT	WT	**70.46 ± 7.37**	4.47 ± 2.35	4.9 ± 2.42	N. D.
16	Sputum	Kw197/13	0.063	0.06	0.5	WT	WT	**64.04 ± 2.34**	3.83 ± 0.95	1.8 ± 0.61	N. D.
22	Urine	Kw3153/14	0.125	0.02	0.5	S113A	WT	**85.92 ± 2.25**	1.11 ± 0.29	4.5 ± 0.45	N. D.
49	Blood	Kw3267/17	0.250	0.19	0.5	WT	WT	**72.76 ± 5.73**	2.01 ± 0.95	2.2 ± 1.00	N. D.
7	Urine	Kw3352/11	1	**32**	**4**	S113A + **G121C**	N313S	0	**9.11 ± 2.65**	**93.6 ± 8.44**	N. D.
27	Urine	Kw135/15	**2**	**32**	**4**	**E105K** + S113A	WT	0	**14.63 ± 3.25**	**94.9 ± 7.35**	N. D.
44	BAL	Kw2327/17	**2**	**32**	**2**	**M93I** + S113A	WT	0	**26.35 ± 1.88**	**93.0 ± 5.63**	N. D.
51	Ear	Kw1075/18	**2**	**32**	**2**	S113A + **H155R**	WT	0	**5.50 ± 0.42**	**95.8 ± 6.91**	N. D.
56	ET	Kw2153/18	0.250	**4**	1	S113A	**S218P**	0	1.83 ± 0.97	**14.6 ± 1.52**	**67.9 ± 2.3**
64	Sputum	Kw1661/19	1	**32**	**2**	S113A + Δ617t, fsm ^d^	WT	**77.93 ± 0.29**	1.00 ± 0.46	1.7 ± 0.58	N. D.
72	BAL	Kw196-11/20	0.25	**32**	**>16**	**L107S** + S113A	WT	0	**10.75 ± 5.18**	**98.1 ± 8.75**	N. D.
73	Urine	Kw20-12/20	**2**	**32**	**>16**	**G90C** + S113A	WT	0	**9.80 ± 2.86**	**96.2 ± 5.92**	N. D.

^a^ BAL, bronchoalveolar lavage; ET, endotracheal aspirate. ^b^ CLSI, reference Clinical Laboratory Standard Institute method; MCN-AM, colorimetric Micronaut-AM assay; MIC, minimum inhibitory concentration; AMB, amphotericin B.; MIC values above the epidemiological cutoff value are shown in bold. ^c^ Mutations in *ERG2* or *ERG3* causing alterations in total cell sterol levels are shown in bold. WT, wild-type sequence as in *C. kefyr* ATCC28838. ^d^ Deletion of ‘t’ nucleotide at position 617 is a frame shift mutation (fsm). ^e^ Sterol values (percentage of total sterols) are presented as mean ± standard deviation and values >5% are shown in bold. ^f^ Fecosterol Plus included fecosterol, ergosta-8-enol, ergosta-8,22-dienol, ergosta-5,8,22-trienol and ergosta-5,8,24(28)-trienol. N. A., Not available; N. D. not detected.

## Data Availability

All the relevant data are present in the manuscript. Additional details can be provided by the corresponding author upon reasonable request.
